# Botanical-chemical formulations enhanced yield and protection against *Bipolaris sorokiniana* in wheat by inducing the expression of pathogenesis-related proteins

**DOI:** 10.1371/journal.pone.0196194

**Published:** 2018-04-30

**Authors:** Rabia Naz, Asia Nosheen, Humaira Yasmin, Asghari Bano, Rumana Keyani

**Affiliations:** 1 Department of Biosciences, COMSATS Institute of Information Technology (CIIT), Chak Shahzad, Islamabad, Pakistan; 2 Department of Biosciences, University of Wah, Wah Cantt, Pakistan; Agroecological Institute, CHINA

## Abstract

Two experiments (pot and field experiments) were conducted in two consecutive years to evaluate the protective effects of botanical-chemical formulations on physiological, biochemical performance and grain yield of wheat inoculated with *Bipolaris sorokiniana*. We compared different formulations comprising *Calotropis procera*, *Jacaranda mimosifolia*, *Thevetia peruviana* extracts, chemical fungicide (mefenoxam) and salicylic acid to modulate the defense system of wheat host plants. Among the selected plant species *J*. *mimosifolia* aqueous and methanolic leaf extracts (1.2% w/v) resulted in 96 to 97% inhibition against *B*. *sorokiniana*. Both in pot and field experiments, among all the formulations of selected plant extracts the combined formulation of JAF2 (*J*. *mimosifolia* 0.6%)+MFF2 (mefenoxam 0.1%) lowered the dose of chemical fungicide required to reduce the leaf spot blotch disease. The same formulation induced resistance in wheat apparently through the accumulation of peroxidase, polyphenol oxidase, protease, acid invertase, chitinase and phenylalanine ammonia lyase. This formulation also stimulated the defense-related gene expression of PR-proteins. The same treatment gave even more increase (48%, 12% and 22%) in no. of grains/spike, grains weight and grain yield, than the MFF1 (mefenoxam 0.2%). We conclude that foliar application of *J*. *mimosifolia* leaf extract with very low dose of chemical fungicide (*J*. *mimosifolia* 0.6%+mefenoxam 0.1%) is a promising approach for the management of leaf blight and spot blotch in wheat.

## Introduction

Spot blotch (foliar blight) caused by *Bipolaris sorokiniana* (Sacc.) Shoem poses a serious and persistent challenge to wheat grown under hot, humid subtropical climate [1]. *B*. *sorokiniana* is a worldwide spread fungus that is particularly aggressive under high relative humidity and temperature associated with imbalanced soil fertility causing severe economic losses in cereal crops every year [2]. Foliar blight has a special significance in South Asia that includes Bangladesh, India and Nepal [3].

In Pakistan wheat crop is affected by many fungal diseases among which, foliar blight/spot blotch is considered to contribute significantly to reduce the average yields of the crop [4]. Earlier studies on foliar blights inciting pathogens in the rice-wheat cropping system of Northern Punjab reported that *Bipolaris sorokiniana* (syn. *Helminthosporium sativum)*, *Drechslera tetramera*, *Pyrenophora tritici-repentis*, *Alternaria alternata* and *Stemphylium* sp., are the major foliar pathogens of wheat [5]. *B*. *sorokiniana* is the most prevalent pathogen in Pakistan causing seedling blight, root rot [6] and leaf blotch of wheat [7].

Chemical fungicides have been found very effective in controlling the fungal diseases of plants [8], but there are some major problems which threaten to limit the continuous use of fungicides. The use of fungicides may cause hazard effects to human health and increase environmental pollution. Therefore, it is important to find alternatives, eco-friendly approaches to control fungal diseases of crop plants. Many researchers have tried to find out safe and economical control of plant diseases using plant extracts [9]. Plant extracts and biofungicide are also available to treat infected seeds as well [10].

Systemic acquired resistance (SAR) is a form of induced resistance that is activated throughout the plant after being exposed to elicitors from virulent, avirulent, or nonpathogenic microbes, or artificial chemical stimuli such as chitosan or salicylic acid (SA) [11, 12].

Stimulation of induced resistance against pathogens is a promising approach for controlling fungal diseases. The use of a chemical elicitor such as salicylic acid (SA) can induce resistance against abiotic and biotic stresses via activation of specific defense responses and defense related enzymes [13]. SA is involved in the induction of expression of specific enzymes catalyzing reactions to form defense compounds such as polyphenols, as well as pathogenesis-related (PR) proteins, especially PR-1 [14, 15].

Plant extracts represents a natural and ecological approach for controlling diseases and provides many distinctive benefits to farmers, as they degrade quickly, have very short pre-harvesting intervals, and reduce the risk of residual effects on food. Natural plant products have been found to be effective in plant disease management and can be safely incorporated as fast-acting and non-pollutive suitable alternative to synthetic fungicides [16]. Our colleague [17], reported the *in vitro* antifungal potential of various plant extracts against several plant pathogens.

Secondary metabolites present in plant extracts such as phenolic compounds are known to contribute in resistance of fungal pathogens infected plants and wound healing via cell wall lignification around the wound area [18]. Martínez [19] reported several factors inducing specific PR proteins and reactive oxygen species (ROS) in the host plant, both of which have antifungal activity. Peroxidases and phenylalanine ammonia lyase (PAL) are the main enzymes involved in phenyl-propanoid metabolism [20]. Specific PR proteins such as chitinases are known to degrade fungal cell walls and provide resistance against fungal pathogens [21].

The main objective of this study was to evaluate *in vitro* and *in vivo* effects of the botanical-chemical fungicide formulations against the *B*. *sorokiniana* in wheat to develop an environmental friendly biofungicide. In different trials on wheat under glasshouse and field conditions our aims were to: 1) compare the effects of formulations using *C*. *procera*, *J*. *mimosifolia*, *T*. *peruviana*, leaf extracts with SA, chemical fungicide (mefenoxam) and Tween-80 (as an emulsifier) in controlling *B*. *sorokiniana*-mediated leaf blight/spot blotch of wheat in both pot and field experiments; 2) monitoring changes in physiological and biochemical responses and in defense-related proteins in wheat; 3) quantify the effects of treatments on the expression of defense-related genes in inoculated wheat leaves; 4) determine the effects of different botanical-chemical formulations and host resistance on grain yield.

## Materials and methods

### Host plant and pathogen material

The leaf spot blotch-susceptible wheat cultivar, Bhakkar 2002, and the pathogen *Bipolaris sorokiniana* (syn. *Helminthosporium sativum)*, were obtained from National Agricultural Research Centre (NARC), Islamabad, Pakistan.

### Preparation of plant extracts and determination of *in vitro* fungicide activity

*Calotropis procera* (Ait.) (Asclepiadaceae), *Jacaranda mimosifolia* D. Don (Bignoniaceae) and *Thevetia peruviana* (Pers.) K. Schum (Apocynaceae) were selected for the experiment based on their *in vitro* antifungal activity against *B*. *sorokiniana*.

The plant species, *Jacaranda mimosifolia* and *Thevetia peruviana* leaves were collected from Quaid-i-Azam University campus, Islamabad while *Calotropis procera* leaves were collected from District Layyah, South Punjab. Collected leaf samples were identified by the National Herbarium, Department of Plant Sciences, Quaid-i-Azam University Islamabad, Pakistan.

Plant leaves were washed thoroughly with distilled water and were shade-dried at room temperature. The dried leaves were uniformly ground using an electric grinder. The powdered plant material (250 g) was extracted for 4 d in 100% methanol and for 24 h in sterile distilled water. The separated extracts were then filtered through Whatman No. 1 filter paper and the methanol filtrate evaporated to dryness using a rotary evaporator at room temperature (30°C). Dried extract was stored in an air-tight container at 4°C until further use. Concentrations of 0.8, 1.0 and 1.2% (w/v) in (separate) methanol and water extracts were used for the *in vitro* antifungal assay.

The agar tube dilution method was used for the determination of antifungal activity of the selected plant extracts (*C*. *procera*, *J*. *mimosifolia* and *T*. *peruviana*) [22]. Samples were prepared by dissolving 12 mg extract in 1 mL of dimethyl sulfoxide (DMSO). Culture medium was prepared by dissolving 6.5 g of Sabouraud dextrose agar per 100 mL distilled water (pH 5.6). A solution (10 mL) of Sabouraud dextrose agar (6.5%) (Merck) was dispensed in screw-capped tubes or cotton-plugged test tubes and was autoclaved at 121°C for 21 min. Tubes were allowed to cool at 50°C and the Sabouraud dextrose agar solution was loaded with 67 μL of extract pipetted from each of the 0.8, 1.0 and 1.2% (w/v) methanol and water extracts. The tubes containing the media were then allowed to solidify in slanting position at room temperature.

Three slants of the extract sample from each concentration of methanol and water extracts were prepared. The tubes containing solidified media and plant extract were inoculated with a 4 mm-diameter piece of inoculum, taken from a 7-day-old culture of the fungus. Slants without extract were used as positive controls (Terbinafine hydrochloride) and negative controls (DMSO or sterile water). The test tubes were incubated at 28°C for 7 d. Cultures were examined twice a week to observe fungal growth during incubation. Mean growth values were obtained after 7 d and were converted to percentage inhibition of mycelial growth in relation to the control using the following formula:

$$\text{MGI}\;\left(\%\right)=\left(\frac{\text{gc}-\text{gt}}{\text{gc}}\right)\times 100$$

Where MGI is minimum growth inhibition, and gc and gt represent mycelial growth in control and treated samples, respectively.

### Preliminary evaluation of fungicides against *Bipolaris sorokiniana* for *in vivo* experiment

The effect of selected fungicides (Ridomil (mefenoxam), Mancozeb, Carboxin) on the radial growth of *B*. *sorokiniana* was determined on PDA medium following the method as described above.

### Preparation of inoculum and treatments for pot and field experiments

Inoculum suspension was prepared by scraping 10–12 d old pathogen culture and dissolving in sterile water. The suspension was incubated at room temperature on shaker and filtered through cheese cloth to remove any pieces of agar and pure conidial culture was obtained. A drop of tween 20 was added to avoid the aggregation of conidia and the concentration was adjusted to 1 × 10^6^ conidia mL^-1^ by diluting with sterile water.

Various treatments were prepared from the leaf extracts of *C*. *procera*, *J*. *mimosifolia*, *T*. *peruviana*, fungicide-Ridomil (45.3% mefenoxam (Syngenta, US), SA (Sigma) and Tween-80 (0.6% v/v). All formulations were packaged in 1-L plastic bottles and stored in the dark at room temperature prior to application.

### Pot experiment

Pot experiments were conducted in two consecutive years (2010–11 & 2011–12). Potting soil (soil and sand at 3:1 w/w); with pH 7.1 and containing the available amount of nutrients Na, K, P, Mg and Ca were 19, 12, 9, 1.8 and 25 μg/g respectively. The soil was filled in sterilized plastic pots (25x40 cm^2^). The surface-sterilized ten seeds were sown in each pot and thinned to six plants per pot after germination. A completely randomized design (CRD) was followed with 18 treatments (listed in Table 1) with three replications in glasshouse with an average temperature of 20–25°C and day length ranging from 10–13 h.

**Table 1:** Symbols used for different formulations tested in pot and field experiments. (1–18 treatments were used for physiological, biochemical and yield parameters, while 6–18 treatments were used for disease assessment parameters).

S/N	Treatments	Symbols used
1.	Healthy Control	Control
2.	*Jacaranda* (1.2%)-Formulation 1	JAF1
3.	*Thevetia* (1.2%)-Formulation	THF1
4.	*Calotropis* (1.2%)-Formulation 1	CAF1
5.	Salicylic acid (4mM) -Formulation 1	SAF1
6.	Infected control with *B*. *sorokiniana*	BS
7.	Mefenoxam (0.2%)+ *B*. *sorokiniana*	MFF1+BS
8.	*Jacaranda* (1.2%)+ *B*. *sorokiniana*-Formulation 1	JAF1+BS
9.	*Thevetia* (1.2%)+ *B*. *sorokiniana*-Formulation 1	THF1+BS
10.	*Calotropis* (1.2%)+ *B*. *sorokiniana*-Formulation 1	CAF1+BS
11.	Salicylic acid (4mM) + *B*. *sorokiniana*-Formulation 1	SAF1+BS
12.	*Jacaranda* (0.6%)+ Mefenoxam (0.1%)+ *B*. *sorokiniana*-Formulation 2	JAF2 + MFF2 + BS
13.	*Thevetia* (0.6%)+ Mefenoxam (0.1%)+ *B*. *sorokiniana*-Formulation 2	THF2 + MFF2 + BS
14.	*Calotropis* (0.6%)+ Mefenoxam (0.1%)+ *B*. *sorokiniana*-Formulation 2	CAF2 + MFF2 + BS
15.	Salicylic acid (2mM) + Mefenoxam (0.1%)+ *B*. *sorokiniana*-Formulation 2	SAF2 + MFF2 + BS
16.	*Jacaranda* (0.6%)+Salicylic acid(2mM) + *B*. *sorokiniana*-Formulation 2	JAF2 + SAF2 + BS
17.	*Thevetia* (0.6%)+Salicylic acid(2mM) + *B*. *sorokiniana*-Formulation 2	THF2 + SAF2 + BS
18.	*Calotropis* (0.6%)+Salicylic acid(2mM) + *B*. *sorokiniana*-Formulation 2	CAF2 + SAF2 + BS

### Field experiment

Field experiment was conducted in the two consecutive years (2010–11 & 2011–12) at wire house of Quaid-i-Azam University, Islamabad (Pakistan) to assess the efficacy of selected plant extracts, SA, synthetic fungicide(mefenoxam) alone and in combinations against leaf spot blotch disease in a randomized complete block design (RCBD) with three replications. The surface sterilized seeds of susceptible wheat cultivar Bhakkar 2002 were sown in plots of 1x1m^2^ in size. Physico-chemical properties of field soil are mentioned in Table 2.

**Table 2:** Physico-chemical properties of the field soil.

Physico-chemical properties of field soil	
Texture class	Sandy clay loam
pH	7.70
E.C	2.13 dSm^-1^
P	6.50 (mg/kg)
Ca^2+^	13.07 (μg/g)
Na^+^	9.91 (μg/g)
Mg^2+^	4.39 (μg/g)
K^+^	10.35 (μg/g)
Cu^2+^	1.39 (μg/g)
Zn^2+^	3.33 (μg/g)
Co^2+^	1.83 (μg/g)
Fe^2+^	9.97 (μg/g)
Cr^3+^	1.43 (μg/g)

### Inoculation of pathogen and application of treatments

Wheat plants were inoculated with the spore suspension of *B*. *sorokiniana* by spraying (10^6^ conidia mL^–1^) at the booting stage in both the pot and field experiments. In pot experiment the moisture was provided after inoculation by covering all the plants with polythene bags and profusely sprayed inside with autoclaved water for better proliferation of the pathogen. In field experiment the plots with inoculated plants were covered with polythene sheets to provide humidity for disease proliferation. Foliar application of plant extract formulations was done after 24 h of pathogen inoculation at high level of humidity. Control plants were sprayed with autoclaved water in both pot and field experiments.

### Sample collection and assessment of disease severity

Plant leaf tissues were harvested from both pot and field experiments at three different stages (3, 6 and 9 d) after the inoculation of *B*. *sorokiniana* to determine changes in defense-related enzymes, protein concentration and total soluble phenolic content. Leaf samples were harvested from randomly selected plants from three replications per treatment at the respective sampling times (during the period 10:00–12:00 A.M.).

After 9 d of inoculation, five plants from each replication/treatment in the pot and field experiments were harvested and scored for disease incidence. The severity symptoms of leaf spot blotch disease were evaluated based on the visual rating scale (Table 3). The development of symptoms was scored on a 0–5 scale based on the affected leaf area.

**Table 3:** Visual rating scale (0–5) for leaf spot blotch.

Infection type	Disease severity symptoms
0	No visible symptoms
1	1–5% visible/noticeable spots on leaves
2	6–20% visible/noticeable spots on leaves
3	21–40% visible/noticeable spots on leaves
4	41–60% visible/noticeable spots on leaves
5	More than 60% visible spots on leaves

Disease incidence was calculated using the following formula: [23]

$$\text{Disease}\;\text{incidence}\;\left(\%\right)=\frac{\text{Number}\;\text{of}\;\text{infected}\;\text{plants}}{\text{Total}\;\text{number}\;\text{of}\;\text{plants}}\times 100$$

The percent disease severity (disease index) was calculated using the formula: [24]

$$\%\;\text{Disease}\;\text{index}\;\left(\text{PDI}\right)=\frac{\text{Disesae}\;\text{index}}{\text{Total}\;\text{infected}\;\text{plants}}\times \frac{100}{5}$$

Diseaseindex=[spotsinscale1]+[spotsinscale2]+….[spotsinscale5]

### SPAD (Soil-Plant Analyses Development) measurement of chlorophyll

Leaf chlorophyll index was measured by SPAD (Minolta Reading SPAD 502). Only completely grown young leaves were used for the measurements (three replicates/treatment).

### Estimation of total protein content

Protein content of the leaves was determined following the method of Lowry, [25] using bovine serum albumin (BSA) as standard. Fresh leaves (0.1 g) were ground in 1 mL of phosphate buffer pH 7.5 with a mortar and pestle and the homogenate was centrifuged at 3,000 rpm for 10 min at room temperature. The supernatant was transferred to test tubes and distilled water added to make a total volume of 1 mL. Alkaline copper sulfate reagent (1 mL) was added and, after shaking for 10 min, 0.1 mL of the Folin’s reagent was added and the mixture was incubated for 30 min. The absorbance of each sample (three replicates/treatment) was recorded at 650 nm against a blank (1.0 mL of 0.5 M sodium hydroxide). The concentration of soluble protein was determined with reference to a standard curve using BSA.

### Total soluble phenol content

Total phenol in wheat leaves was extracted as described by Hsu, [26] with some modifications. Fresh leaves (0.625 g) were homogenized in 10 mL of methanol and kept overnight. The filtrate was diluted to 100 mL, and served as a stock solution. Following the reported method [27], Stock solution (200 μL) was added to 1.4 mL of distilled water and 0.1 mL of 50% Folin- Ciocalteu phenol reagent. After 3 min, 20% (w/v) sodium carbonate (0.3%) was added. The mixture was allowed to stand for 2 h. After gentle vortex, the absorbance was noted at 765 nm. Total soluble phenol content was standardized against gallic acid.

### Determination of acid invertase (AI) activity

Acid invertase (AI) activity of leaves was measured [28], with some modifications. Leaf segments (0.25 g) were excised after inoculation of the pathogen from control and infected leaf tissues, immersed in ice-cold ethyl acetate for 20 min and finally washed in ice-cold distilled water. Each sample was incubated in 0.1 M sodium phosphate buffer (pH 5.6), 0.5 M sucrose and distilled water in a water bath at 30°C for 60 min. The remaining water on the leaf surface was blotted with filter paper. Each leaf sample was transferred into a 20-mL vial containing 2 mL of 0.5 M sucrose, 2 mL of 0.1 M sodium phosphate buffer (pH. 5.6) and 6 mL of double-distilled water. The vials were incubated in a shaking water bath at 30°C for 60 min. AI activity was measured by reading the absorbance of each sample (three replicates per treatment) at 280 nm in a spectrophotometer (HITACHI Model: U-1100 573×415).

### Determination of protease activity

Protease activity was assessed by the method of McDonald and Chen [29]. Leaf tissue (100 mg) was incubated with 4 mL of the substrate (1% casein in 0.1 M sodium citrate buffer of pH 7.0) for 1 h at 30°C. Five millilitres of 5% trichloroacetic acid (TCA) was added to precipitate the residual protein. The precipitate was allowed to settle for 30 min and the contents of the tubes were filtered through filter paper (Whatman No. 40). After filtration, a 1-mL aliquot of the filtrate was mixed with 5 mL of alkaline reagent mixture, which was prepared by mixing 100 mL of sodium carbonate (2% w/v), 1 mL of sodium potassium tartrate (2.7% w/v) and 1% (w/v) copper sulfate. Two millilitres of 1 M sodium hydroxide was then added to make the solution alkaline. After a minimum of 10 min, Folin-Ciocalteu-phenol reagent (0.5 mL) was added and the contents were mixed. The absorbance of the blue color produced in each sample (three replicates per treatment) was measured at 660 nm after 30 min using a spectrophotometer. One unit of protease activity was defined as the amount of enzyme required to produce an increase in optical density at 660 nm of 0.1 h^-1^ at 30°C at pH 7.0.

### Estimation of polyphenol oxidase (PPO) activity

The PPO activity was determined following the method Kar and Mishra [30], with some modifications. Leaf tissue (1 g) was homogenized in 2 mL of 0.1 M sodium phosphate buffer (pH 6.5) and centrifuged at 10,000 × g for 25 min at 4°C. The supernatant was used as an enzyme extract. The 3-mL reaction mixture contained 25 mM phosphate buffer (pH 6.8), 0.1 mM pyrogallol, 0.1 mL enzyme extract and blank without pyrogallol. The absorbance of each sample (three replicates/treatments) was recorded at 420 nm.

### Estimation of peroxidase activity (POD)

Leaf tissue (1 g) was homogenized in 5 mL of 0.05 M phosphate buffer (pH 7.0) containing 10% polyvinylpyrrolidone (Sigma) and 0.1 M ethylene diaminetetraacetic acid (EDTA) (Sigma), and centrifuged at 14,000 rpm for 20 min at 4°C. The supernatant was used for the POD assay [31]. POD activity was measured by the method of Vetter, et al. [32]. The assay mixture contained 0.1 mL enzyme extract, 1.35 mL 0.1 M mM MES buffer (pH 5.5), 0.05% H_2_O_2_ and 0.1% phenylenediamine. Changes in the absorbance in each sample from three replicates per treatment were recorded at 485 nm for 3 min.

### Estimation of chitinase activity

Chitinase activity was determined by colorimetric assay using the purple dye-labeled biopolymeric substrate, CM chitin-RBV (Loewe Biochemical, Germany) [33]. CM-chitin-RBV (200 μL of 2 mg/mL) was mixed with 300 μL of leaf protein extract and 300 μL of 10 mM Tris-HCl, pH 7.5, containing 1% Triton X-100. The mixture was incubated at 37°C for 3 h. The reaction was stopped by the addition of 200 mL of 2 M HCl. Samples were cooled on ice for 15 min and then centrifuged at 16,000 × *g* for 10 min to remove the non-degraded substrate. The supernatant was collected from three replicates per treatment and the absorbance measured at 550 nm. One unit of chitinase activity represented an increase of absorbance of 0.1 at 550 nm [34, 35].

### Estimation of phenylalanine ammonia lyase (PAL) activity

The PAL enzyme was extracted and partially purified following the method Suzuki et al. [36]. Fresh leaves (1 g) were ground at 4°C in 5 mL of 0.1 M sodium borate buffer (pH 8.8). Homogenates were centrifuged at 12,000 × *g* for 15 min at 4°C and the supernatant was used as the enzyme extract. Reaction mixtures consisting of 500 μL sodium borate buffer (pH 8.7) and 250 μL enzyme extracts were pre-incubated for 5 min at 40°C and the reaction was started after the addition of 300 μL of 50 mM l-phenylalanine (Sigma-Aldrich). After incubation for 1 h at 40°C, the reaction was stopped by adding 50 μL of 5 N HCl. The reaction mixture was centrifuged again (12,000 × *g* for 15 min) prior to injection into an HPLC (Shimadzu, C-R4A Chromatopac; SCL-6B system controller) featuring a Zorbax SB-C18 analytical column (4.6 × 150 mm, 5 μm particle size, Agilent, Germany) and a U.V. detector at room temperature. The mobile phase consisted of 57% acetonitrile in water with a flow rate of 0.5 mL min^−1^. Detection of *trans*-cinnamic acid (*t*-CA) in all the treatments (three replicates) was based on retention time and performed at 275 nm. The activity of PAL was expressed as nmol *t*-CA min^−1^ g^−1^ of fresh mass in relation to the peak area of a *t*-CA standard solution (1 mg/100 mL sodium borate buffer, pH 8.7).

### RNA extraction and RT-qPCR

RNA was extracted from leaves using the Isolate RNA Plant Kit (Bioline) according to the manufacturer’s instructions and quantified in a NanoDrop-ND1000 spectrophotometer (Thermo Fisher Scientific Inc.) at a 1:10 (v/v) dilution. Purified RNA was used as template to synthesize cDNA using the Tetro cDNA Synthesis Kit (Bioline). The reaction mixture contained Ribosafe RNase inhibitor (1 μL), dNTP mix (1 μL), oligo (dT)_18_ (1 μL), Tetro reverse transcriptase (1 μL), 5× RT buffer and DEPC-treated water (to 20 μL), incubated at 37°C for 40 min and stored at -20°C.

Four genes involved in wheat plant responses to infection were selected for RT-qPCR analysis (primers in Table A in S1 Text). Real-time reactions were performed using the SensiFAST^™^ SYBR Bo-ROX Kit (BioLine). The reaction consisted of 2.0 μL cDNA (30 ng) and a solution containing 3.2 μL DEPC-treated water, 0.4 μL forward primer (10 μM), 0.4 μL reverse primer (10 μM) and 5 μL 2× SensiFAST^™^ SYBR Bo-ROX mix. For each sample, three independent biological replicates were used (each with three technical replicates), including negative controls in which cDNA was substituted by the same volume of water. A Bio-Rad CX96 thermocycler was used with the following conditions: one cycle of 95°C for 2 min; one cycle of 95°C for 5 s; one cycle of 60°C for 25 s; and 39 cycles of 95°C for 5 s and 60°C for 25 s. RT-qPCR data was analyzed using BioRad CFX Manager (3.0) software.

### Grain yield

Treated and untreated wheat plants grown in fields in two consecutive years were harvested on 13 April 2011 and 15 April 2012, respectively. The spikes were threshed, number of grains per spike were counted, weighed and yield estimated in g/m^2^; grain yield was then converted to kg/ha.

### Statistical analysis

Both pot and field experiments were performed twice. Analyses of the data generated from both repeated experiments exhibited no significant interaction between the two tests run for any of the treatments; therefore, results from duplicate tests were combined for the final analysis and were expressed as ± standard deviation of the mean (SD). The data were then subjected to ANOVA and statistical analysis using Statistix version 8.1. Comparisons among mean values of treatments were made by Least Significant Difference (LSD) to test significant differences at P< 0.05 [37].

## Results

### *In vitro* antifungal potential of selected plant extracts and synthetic fungicides against *B*. *sorokiniana*

Both the methanolic and aqueous leaf extracts of the selected plant species (at concentrations of 0.8, 1.0 and 1.2%) resulted in the inhibition of mycelial growth of *B*. *sorokiniana* (Table 4). Methanolic and water extracts of *Jacaranda mimosifolia* at the concentration of 1.2% showed maximum significant inhibition (97 and 96%) that was followed by *Thevetia peruviana* and *Calotropis procera* (90, 83% and 81, 75%, respectively) compared to the respective positive controls.

**Table 4:** *In vitro* antifungal activity of selected plant extracts against *Bipolaris sorokiniana*.

Treatment	*Bipolaris sorokiniana* growth (cm)		
	Methanol extract		
	0.8%	1.0%	1.2%
*Calotropis procera*	2.60^c^±0.06	1.97^c^±0.12	1.7^b^±0.37
*Jacaranda mimosifolia*	0.67^e^±0.05	0.57^f^±0.05	0.33^d^±0.05
*Thevetia peruviana*	2.40^c^±0.06	1.90^c^±0.06	1.4^b^±0.26
Positive control	0.77^d^±0.12	0.77^e^±0.12	0.77^c^±0.12
Negative control	9.8^b^±0.05	10.00^a^±0.05	9.80^a^±0.03
	**Aqueous extract**		
*Calotropis procera*	3.27^c^±0.07	3.77^b^±0.03	2.5^b^±0.37
*Jacaranda mimosifolia*	0.73^de^±0.05	0.60^e^±0.1	0.43^b^±0.05
*Thevetia peruviana*	2.53^c^±0.32	2.87^b^±0.18	1.9^b^±0.61
Positive control	0.83^d^±0.05	0.83^d^±0.05	0.83^c^±0.05
Negative control	10.00^a^±0.08	10.00^a^±0.05	10.00^a^±0.05

Data represent mean ± standard deviation (SD) of three replicates.

All mean values in the same column with different letters are significantly different from each other at P< 0.05.

Positive control: Terbinafine. Negative control for methanolic extracts: DMSO. Negative control for aqueous extracts: sterile distilled water.

Three selected synthetic fungicides carboxin, mancozeb and mefenoxam (at concentration of 0.5, 0.1, 0.2%) inhibited the mycelial growth of *B*. *sorokiniana* (Table 5). Mefenoxam (Ridomil) exhibited the maximum significant inhibition 80% that was followed by mancozeb (74%) and carboxin (64%) at the concentration of 0.2%, therefore it was selected for the *in vivo* experiment.

**Table 5:** *In vitro* antifungal potential of selected synthetic fungicides against *Bipolaris sorokiniana*.

Treatments	*Bipolaris sorokiniana* (Mycelial growth (cm))		
	0.50%	0.10%	0.20%
Carboxin	5.51^b^±0.92	4.88^b^±0.81	3.59^b^±0.57
Mancozeb	4.14^b^±0.76	3.37^c^±0.69	2.63^c^±0.61
Mefenoxam	3.50^c^±0.71	2.90^d^±0.54	1.97^d^±0.54
Negative control	10.00^a^±0.71	9.80^a^±0.69	10.00^a^±0.62

Data represent mean ± standard deviation (SD) of three replicates.

All mean values in the same column with different letters are significantly different from each other at P< 0.05. Negative control: sterile distilled water.

### Effect of botanical-chemical formulations on disease assessment parameters

In both pot and field experiments, chlorophyll, protein contents and defense-related enzyme activities were significantly increased at 9 d after *B*. *sorokiniana* inoculation.

**Fig 1:**
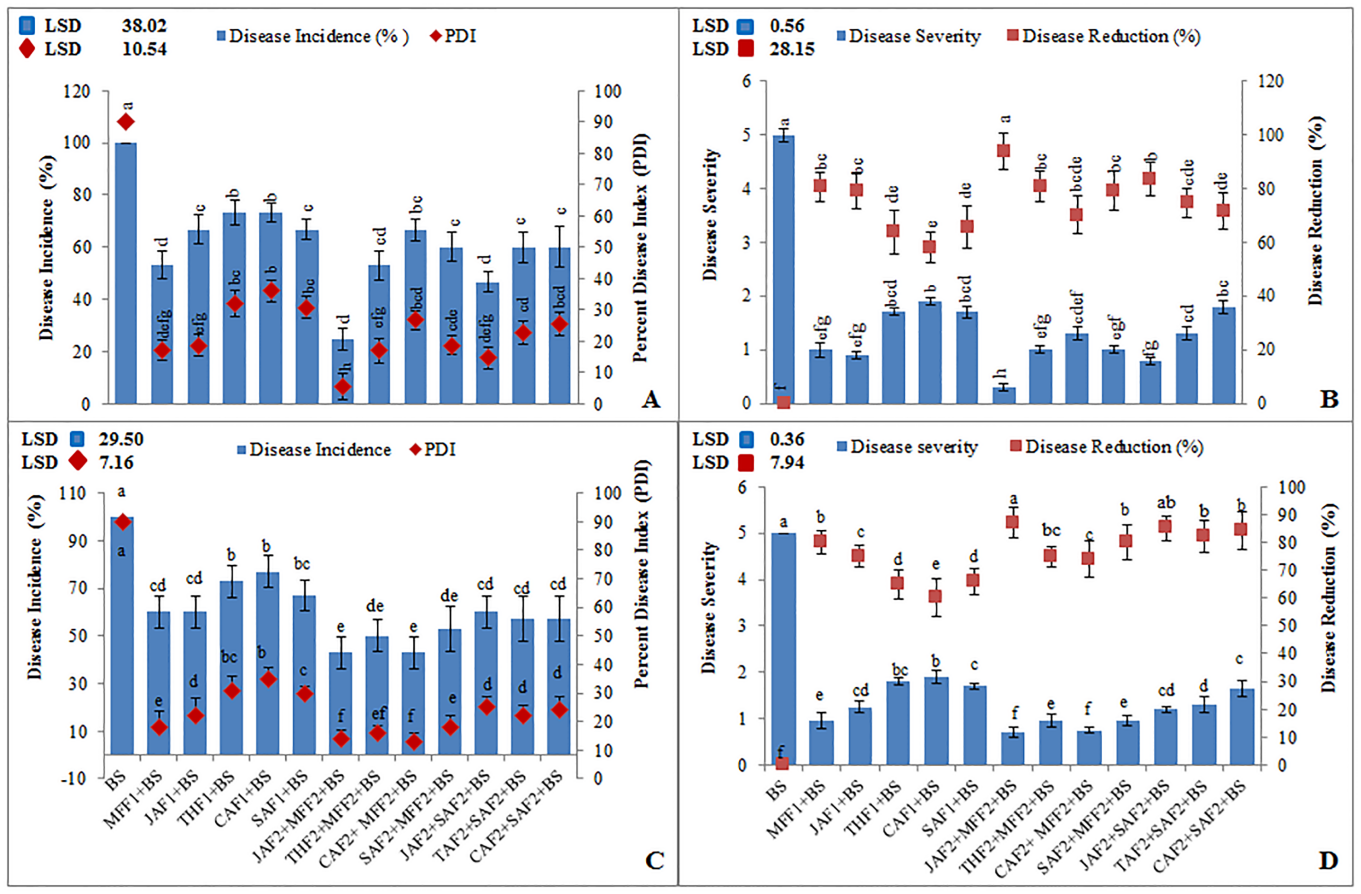
Effect of botanical formulations on disease incidence, percent disease index, disease severity and disease reduction in wheat inoculated with *B*. *sorokiniana* (9 d after inoculation). (A & B) pot experiment under axenic conditions (C & D) field experiment. Data represent means of two years’ pooled experiments with three replicates for both pot and field experiments. Data are expressed as mean ± standard deviation (SD) of three replicates. LSD indicates least significant differences and mean values with different letters on the bars are statistically significant from each other according to the *LSD* test at P< 0.05. See Table 1 for abbreviations.

In the pot experiment, different leaf extract formulations JAF1, THF1, CAF1, SAF1 and MFF1 (*J*. *mimosifolia* 1.2%, *T*. *peruviana* 1.2%, *C*. *procera* 1.2%, SA 4mM and fungicide mefenoxam 0.2%, respectively) each separately, resulted in a significant disease reduction in leaf blotch ranging from 58–94% and disease incidence ranging from 25–73% over the infected control at P<0.05 (Fig 1A and 1B). While in the combined formulations (JAF2+SAF2 and JAF2+MFF2), *J*. *mimosifolia* extract enhanced the effect of the SA and fungicide, reducing the leaf spot blotch disease incidence by 25 and 47%, respectively, and giving a disease reduction by 94 and 84%, respectively, as compared to the infected control. Among all the treatments, the combined formulation JAF2 (*J*. *mimosifolia* 0.6%) + MFF2 (mefenoxam 0.1%) resulted in the maximum reduction (94%), which was 29% and 13% more effective in decreasing the disease incidence and increasing the disease reduction, respectively, than MFF1.

In the field experiment, JAF2, THF2 (*T*. *peruviana* 0.6%) and CAF2 (*C*. *procera* 0.6%) in combination with MFF2, resulted in reduced leaf blotch disease incidence by 57, 50, 57% respectively, and giving a disease reduction by 87, 75 and 74%, respectively, as compared to the infected control (Fig 1C and 1D). While the combined applications, JAF2, THF2 and CAF2 with SAF2 (salicylic acid 2mM) enhanced the effects of SA, reducing the disease incidence by 50 and 54% respectively, and giving a disease reduction by 85, 82 and 84%, respectively, as compared to the infected control.

### Effects of botanical-chemical formulations on the chlorophyll, total protein and phenolic content in wheat leaves

In pot experiments, single applications of JAF1, THF1, CAF1 and foliar spray of SAF1 on uninoculated (uninfected) wheat plants resulted in an increase in chlorophyll content by 39, 25, 26 and 27%, respectively, protein content by 21, 18, 16 and 22%, respectively and phenolic content by 45, 25, 34 and 40%, respectively.

**Fig 2:**
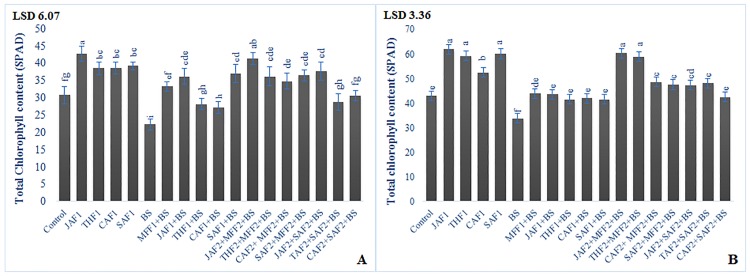
Effect of botanical formulations on chlorophyll contents of wheat leaves from plants inoculated with *B*. *sorokiniana* (9 d after inoculation). **(A)** pot experiment, (**B)** field experiment.

**Fig 3:**
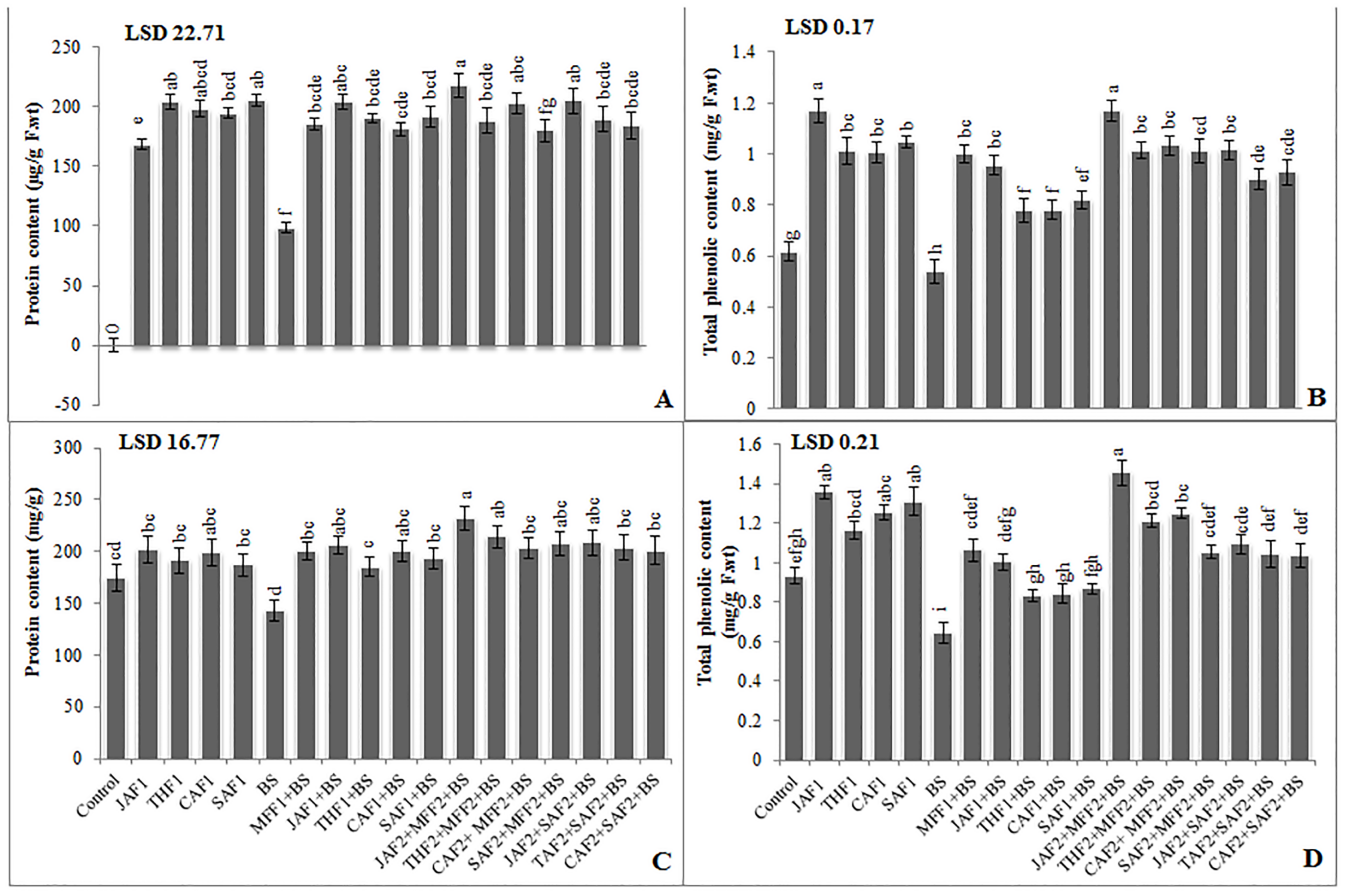
Effect of botanical formulations on protein and phenolic contents of wheat leaves from plants inoculated with *B*. *sorokiniana* (9 d after inoculation). **(A)** Protein content (pot experiment), (**B)** Total soluble phenolic content (pot experiment), (**C)** Protein content (field experiment), (**D)** Total soluble phenolic content (field experiment). Data expressed as ± standard deviation of mean (SD) of three replicates. LSD indicates least significant differences and mean values with different letters on the bars are statistically significant from each other according to the *LSD* test at P< 0.05. See Table 1 for abbreviations.

In the pot experiment, inoculation of wheat plants with *B*. *sorokiniana* resulted in 28, 42 and 31% decrease in chlorophyll (Fig 2A), protein and phenolic content, respectively, as compared to the infected control (Fig 3A and 3B). The MFF1 significantly increased the leaf chlorophyll, protein and phenolic content by 49, 89 and 86%, respectively. Applications of JAF1, THF1, CAF1 and SAF1 in wheat inoculated plants increased the chlorophyll content by 62, 26, 21, 66%, respectively, total protein content by 107, 93, 84, 95%, respectively and phenolic content by 77, 45, 45 and 52% respectively, as compared to the infected control. The maximum increase of 85, 122 and 117% in chlorophyll, protein and phenolic content, respectively, was exhibited by the combined formulations of JAF2+ MFF2. In the field conditions, *B*. *sorokiniana* infection exhibited a significant decrease in chlorophyll, protein and phenolic content by 21, 19 and 31%, respectively, as compared to the infected control (Figs 2B, 3C and 3D). The fungicide treatment MFF1 significantly increased the chlorophyll, protein and phenolic content by 30, 41 and 65%, respectively as compared to infected control. Single application of JAF1, THF1, CAF1 and SAF1 increased the chlorophyll content by 29, 23, 24, 23%, respectively, protein content by 44, 30, 40, 36%, respectively and phenolic content by 56, 29, 31 and 35%, respectively, as compared to the infected control. The maximum increase of 78, 63 and 126% in chlorophyll, protein and phenolic content, respectively, was resulted in the combined application of JAF2+MFF2.

### Effects of botanical-chemical formulations on the activity of defense-related enzyme and pathogenesis-related proteins in wheat leaves

Inoculation of *B*. *sorokiniana* stimulated activities of all the defense-related enzymes tested in all treatments as compared to the uninoculated control.

**Fig 4:**
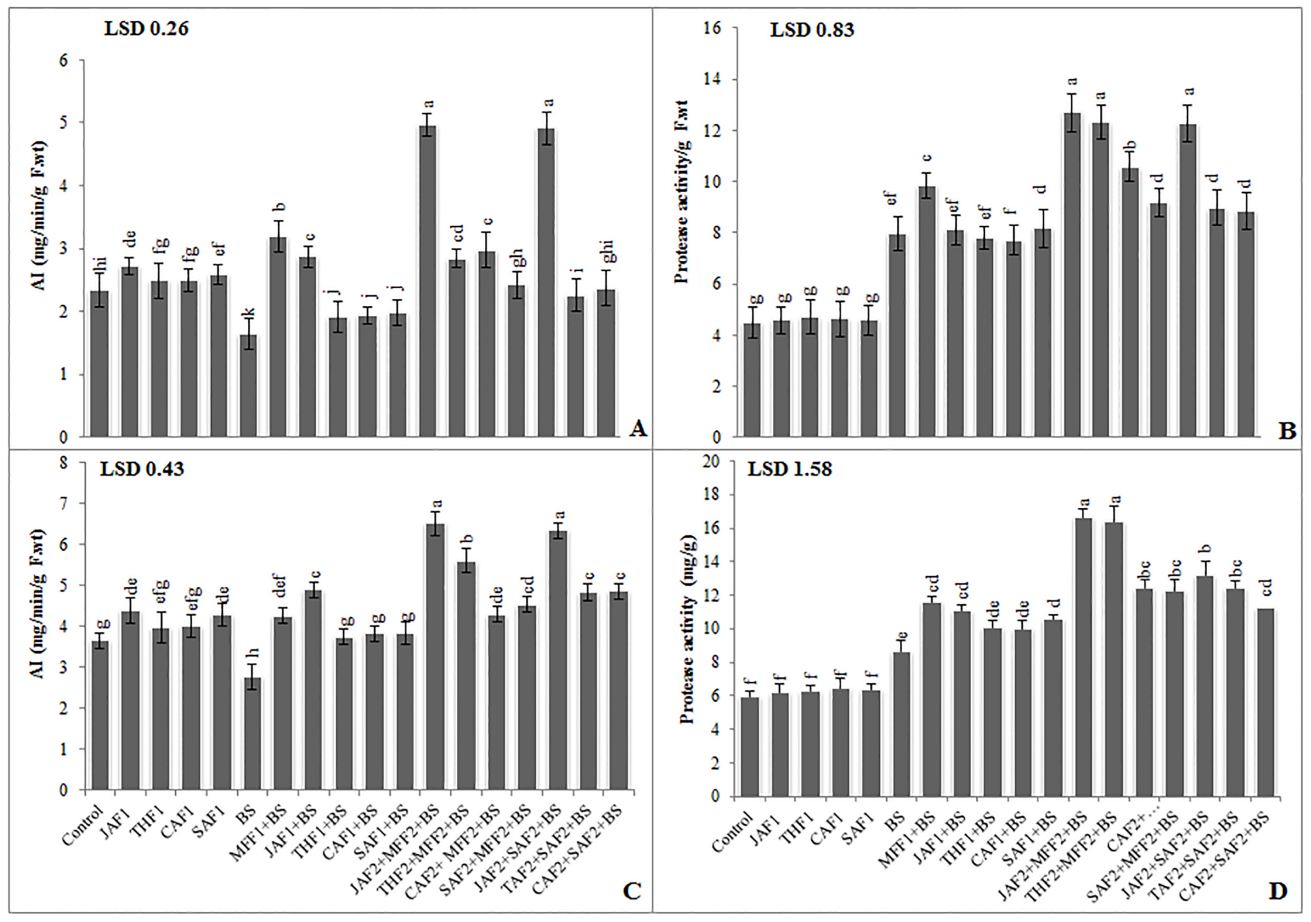
Effect of botanical formulations on acid invertase (AI) and protease activities of wheat leaves from plants inoculated with *B*. *sorokiniana* (9 d after inoculation). **(A)** Acid invertase activity (pot experiment), (**B)** protease activity (pot experiment), (**C)** acid invertase activity (field experiment), (**D)** protease activity (field experiment). Data expressed as ± standard deviation of mean (SD) of three replicates. LSD indicates least significant differences and mean values with different letters on the bars are statistically significantly different from each other according to the *LSD* test at P< 0.05. See Table 1 for abbreviations.

In the pot experiment, single application of JAF1, THF1, CAF1 and foliar spray of SAF1 on uninfected wheat plants resulted in a non-significant increase in AI activity ranging from 7–17%, while non-significant effect on protease activity was observed as compared to control. Acid invertase (AI) and protease activities in the leaves were higher in inoculated plants compared with the untreated plants (Fig 4A and 4B). The fungicide MFF1 application significantly increased the AI and protease activity by 94% and 24%, respectively, as compared with the infected control. Single applications of JAF1, THF1, CAF1 and SAF1 on the inoculated plants increased the AI activity by 75, 17, 18, 21% respectively and showed non-significant increase in protease activity as compared to the infected control. The combined formulation of JAF2+MFF2 resulted in the maximum increase (202% and 59%) in AI and protease activity. In the field, *B*. *sorokiniana* exhibited a significant decrease in AI (25%) and increase in protease activity (45%), compared to the control (Fig 4C and 4D). The fungicide MFF1 treatment significantly increased the AI and protease by 54% and 33%, respectively. Single application of JAF1, THF1, CAF1 and SAF1 increased the AI (78, 36, 38 and 39%, respectively) and protease activity (28, 16, 15 and 22%, respectively) as compared to the infected control. The combined application of JAF2+MFF2 led to the maximum increase of 136% and 92% in AI and protease activity, respectively.

**Fig 5:**
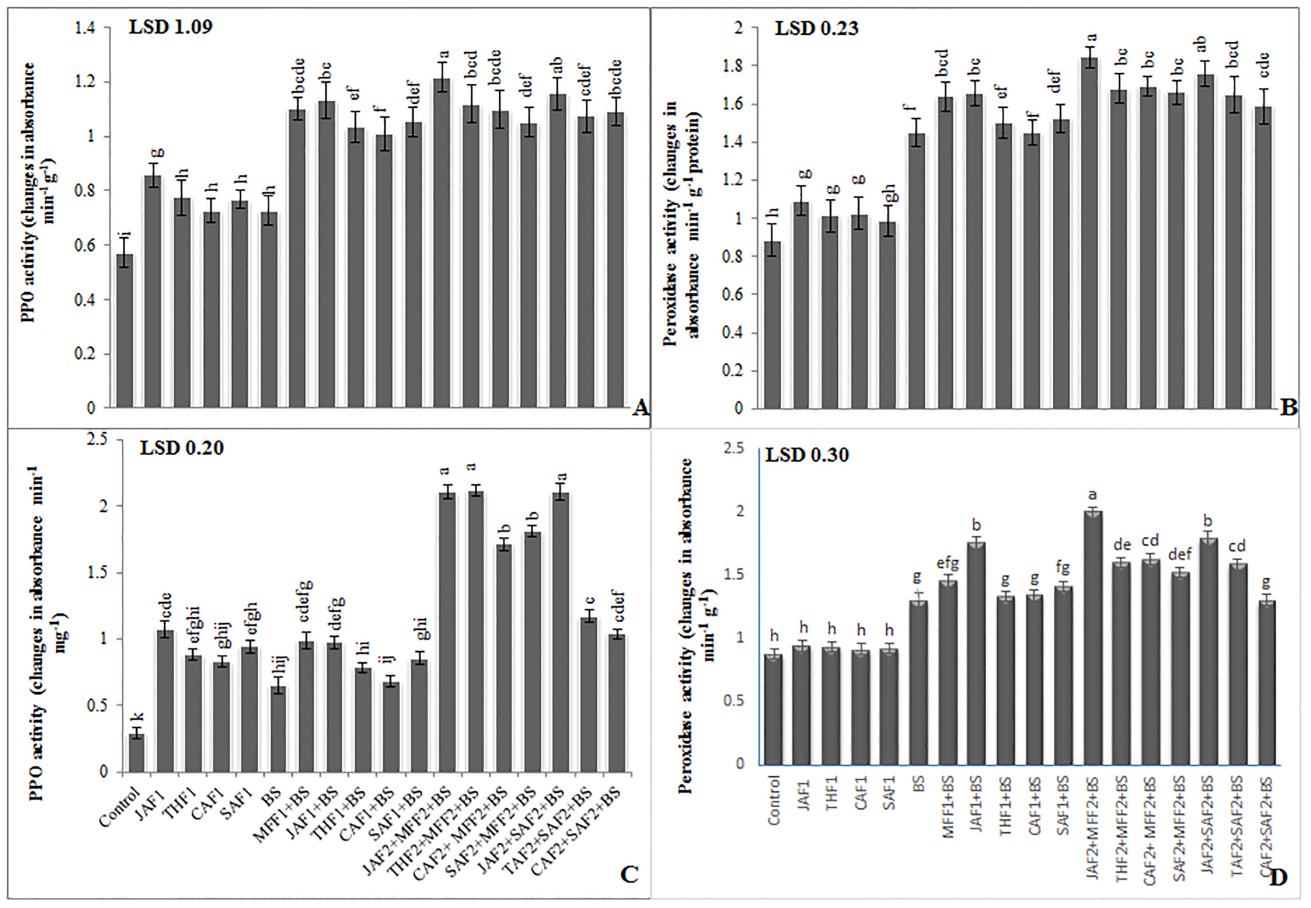
Effect of botanical formulations on polyphenol oxidase (PPO) and peroxidase activities of wheat leaves from plants inoculated with *B*. *sorokiniana* (9 d after inoculation). **(A)** PPO activity (pot experiment), (**B)** peroxidase activity (pot experiment), (**C)** PPO activity (field experiment), (**D)** peroxidase activity (field experiment). Data expressed as ± standard deviation of mean (SD) of three replicates. LSD indicates least significant differences and mean values with different letters on the bars are statistically significantly different from each other according to the *LSD* test at P< 0.05. See Table 1 for abbreviations.

Inoculation of *B*. *sorokiniana*, in both pot and field experiments, induced higher PPO and POD activity in the treated plants than in untreated controls (Fig 5). In the pot experiment, fungicide MFF1 application significantly increased the leaf PPO and POD activities by 52% and 13%, respectively, compared with the infected control (Fig 5A and 5B). Single application of JAF1, THF1, CAF1 and SAF1 increased the leaf PPO (56, 42, 39 and 45%, respectively) and POD (14, 4, 0.3 and 5%, respectively) activities as compared to the infected control. While the combined application of JAF2+MFF2 resulted in maximum increase of 67% and 27% in PPO and POD activities, respectively. In the field experiment, inoculation with *B*. *sorokiniana* showed a significant increase (122% and 69%) in PPO and POD activities, respectively as compared to the infected control (Fig 5C and 5D). The fungicide treatment MFF1 significantly increased the PPO and POD activity by 53 and 12%, respectively. Single applications of JAF1, THF1, CAF1 and SAF1 increased the PPO activity by 50, 21, 14, 32%, respectively and POD activity by 34, 2.4, 3 and 8%, respectively, as compared with the infected control. The combined application of JAF2+MFF2 resulted in a maximum increase of 224 and 53% in PPO and POD activity, respectively.

**Fig 6:**
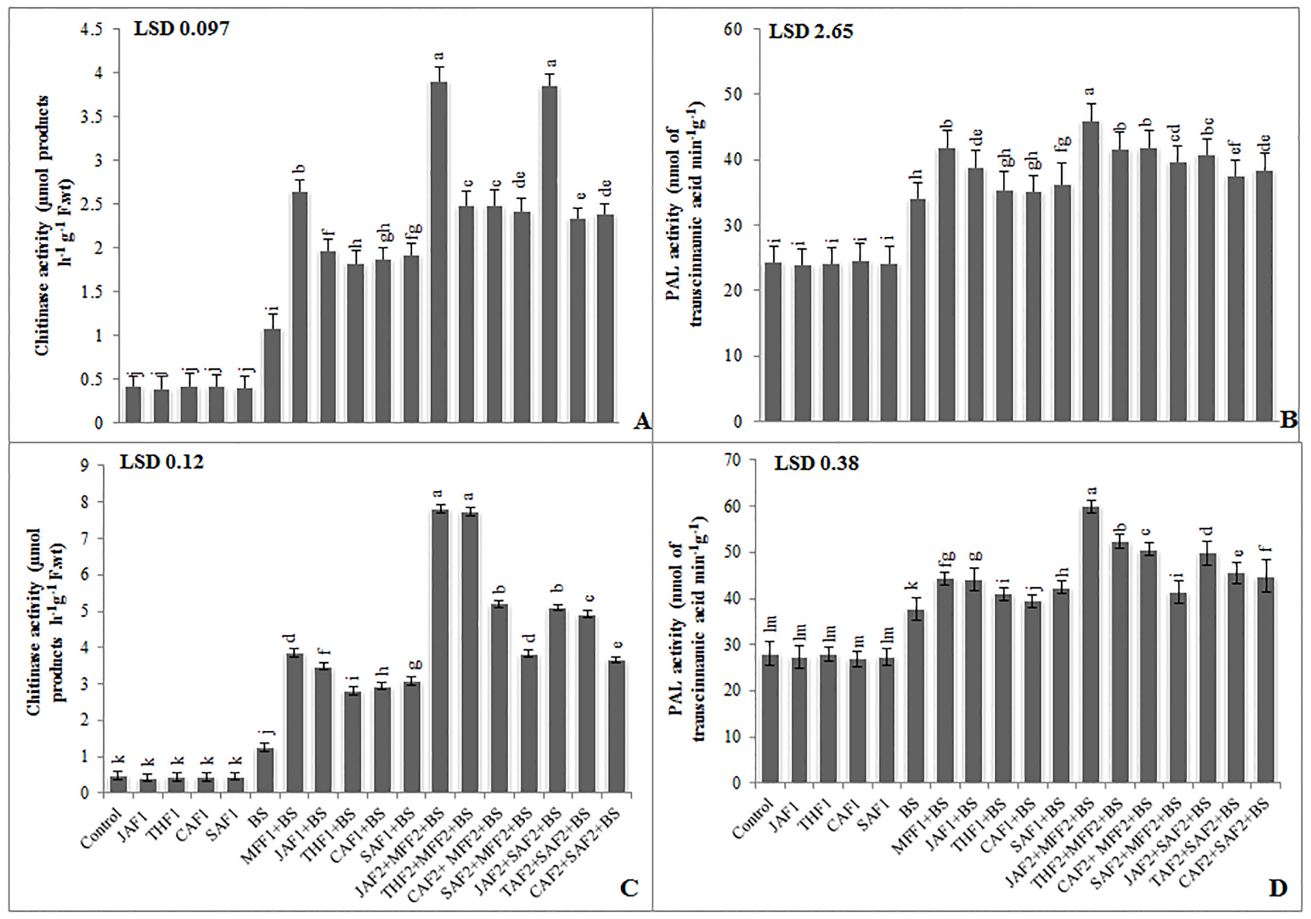
Effect of botanical formulations on chitinase and PAL activities of wheat leaves from plants inoculated with *B*. *sorokiniana* (9 d after inoculation). **(A)** chitinase activity (pot experiment), (**B)** PAL activity (pot experiment), (**C)** chitinase activity (field experiment), (**D)** PAL activity (field experiment). Data expressed as ± standard deviation of mean (SD) of three replicates. LSD indicates least significant differences and mean values with different letters on the bars are statistically significantly different from each other according to the *LSD* test at P< 0.05. See Table 1 for abbreviations.

In the pot experiment, infection of wheat plants with *B*. *sorokiniana* was associated with a 163% and 39% increase in chitinase and PAL activity, respectively, as compared to the infected control (Fig 6A and 6B). Single application of MFF1 significantly increased the leaf chitinase and PAL activity by 144% and 23%, respectively. The JAF1, THF1, CAF1 and SAF1 increased the chitinase acidity by 81, 68, 80, 78%, respectively and PAL activity by 14, 4, 3, and 6% respectively, as compared to the infected control. While the combined application of JAF2+MFF2 resulted in the maximum increase of 313% and 38% in chitinase and PAL activity, respectively. In the field experiment, *B*. *sorokiniana* infection exhibited a significant increase in chitinase and PAL activity by 162% and 35%, respectively, as compared to the infected control (Fig 6C and 6D). The fungicide treatment MFF1 significantly increased the chitinase and PAL activity by 205% and 18%, respectively as compared to infected control. Application of JAF1, THF1, CAF1 and SAF1 treatments increased the chitinase activity by 176, 122, 133, 144%, respectively and PAL activity by 17, 9, 5 and 12%, respectively, as compared to the infected control. The maximum increase of 521% and 39% in chitinase and PAL activity, respectively, was given by the combined application of JAF2+MFF2.

### Effects of botanical-chemical formulations on the differential expression of pathogen-related genes at transcript level

**Fig 7:**
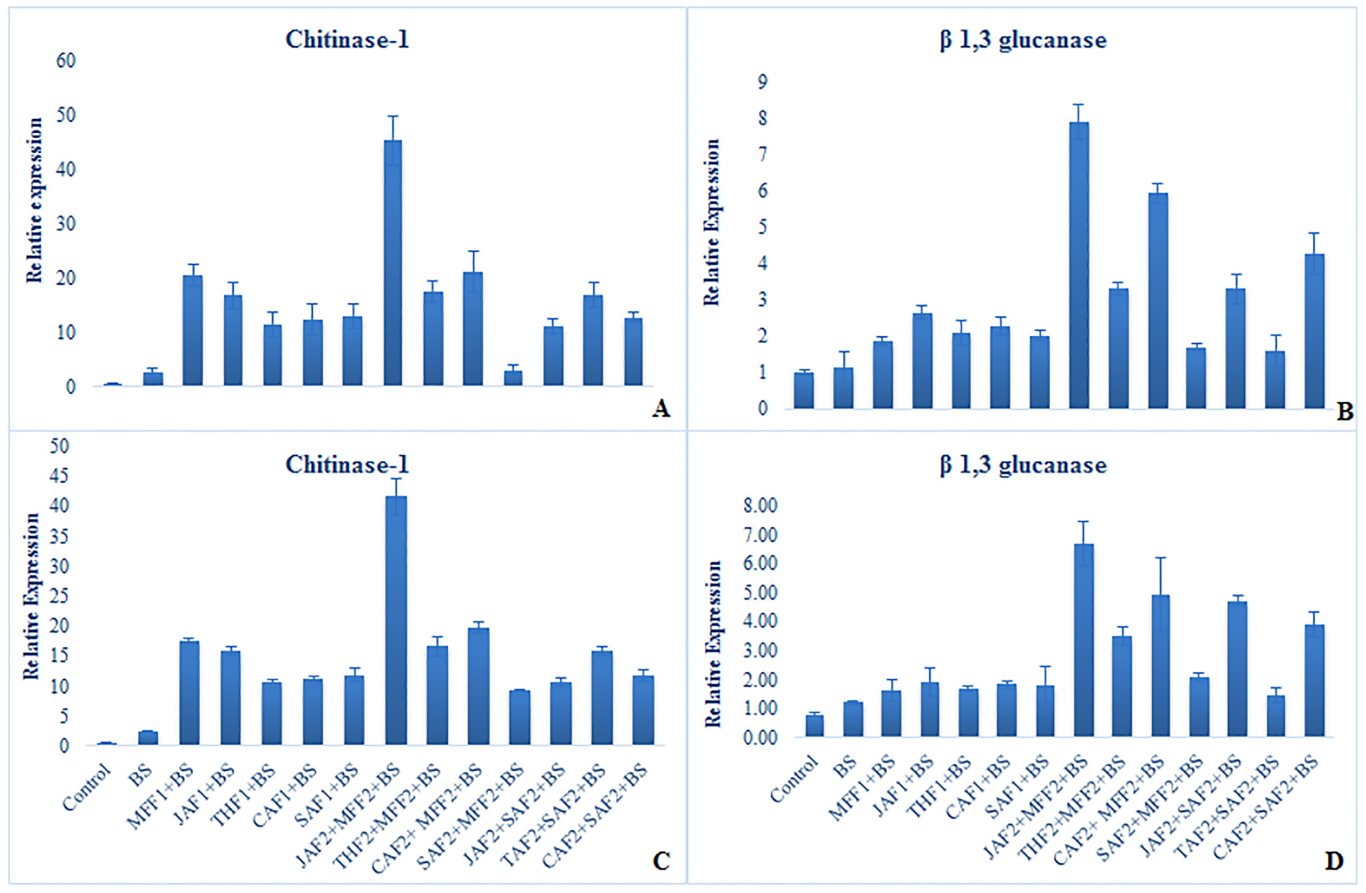
Effect of botanical formulations on relative expression profiles of selected genes 48 h after inoculation of wheat leaves with *B*. *sorokiniana*. Relative transcript abundance was determined using RT-qPCR. Data represent means with standard errors (n = 3). Labeling is explained in Table 1. (**A**) relative expression profiles of chitinase gene (pot experiment), (**B)** relative expression profiles of glucanase gene (pot experiment), (**C)** relative expression profiles of chitinase (field experiment), (**D)** relative expression profiles of glucanase genes (field experiment). Data represent means of two years’ pooled experiments for both pot and field experiments.

**Fig 8:**
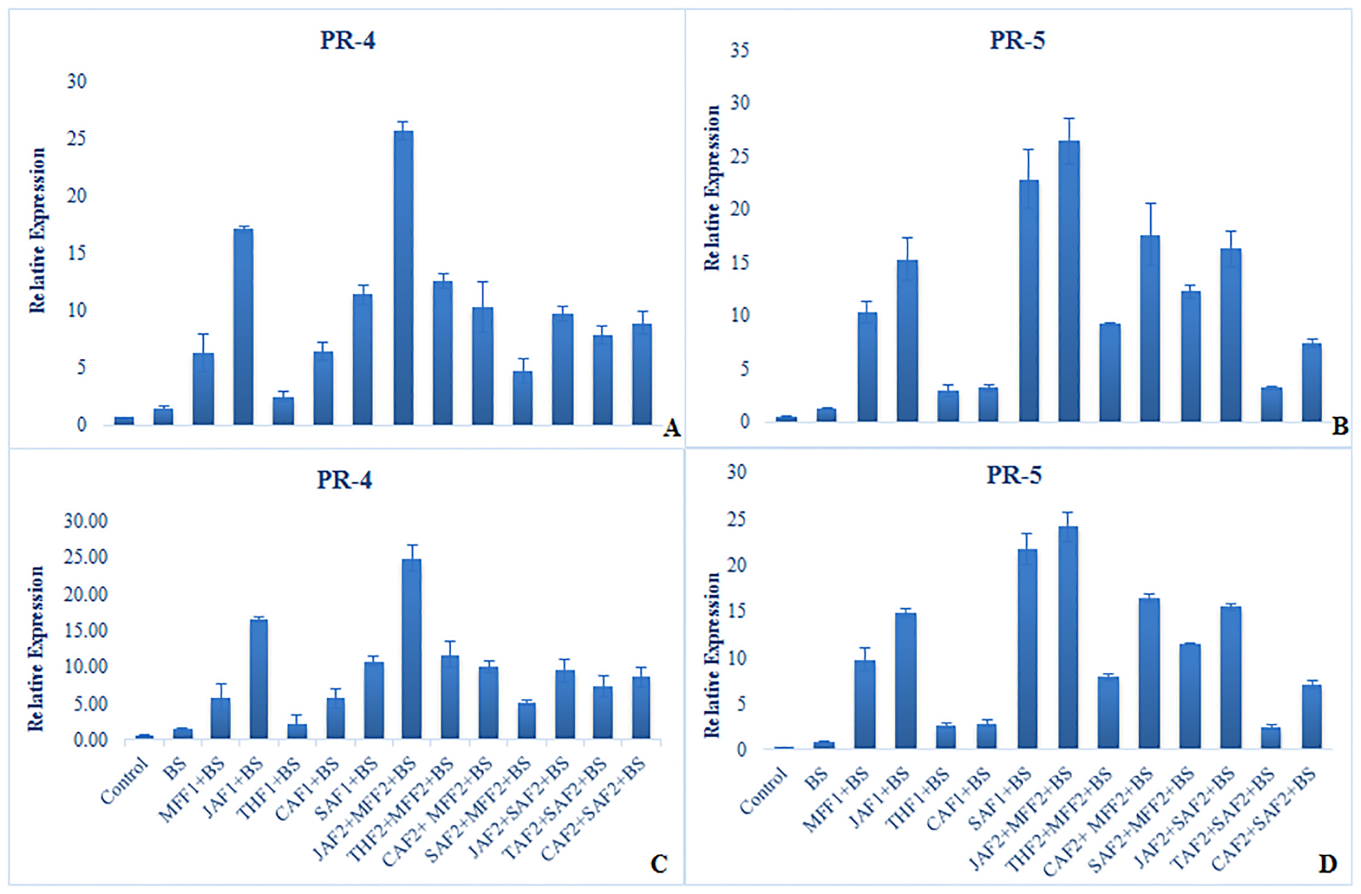
Effect of botanical formulations on relative expression profiles of selected genes 48 h after inoculation of wheat leaves with *B*. *sorokiniana*. Relative transcript abundance was determined using RT-qPCR. Data represent means with standard errors (n = 3). Labeling is explained in Table 1. **(A**) relative expression profiles of *PR-4* gene (pot experiment), (**B**) relative expression profiles of *PR-5* gene (pot experiment), (**C**) relative expression profiles of *PR-4* gene (field experiment), (**D)** relative expression profiles of *PR-5* genes (field experiment). Data represent means of two years’ pooled experiments for both pot and field experiments.

Expression patterns of defense-related genes were analyzed using RT-qPCR (Figs 7 & 8) in the wheat leaves treated with plant extract formulations and inoculated with *B*. *sorokiniana* in both pot and field experiments. All the formulations resulted in substantial up-regulation of the selected genes in response to *B*. *sorokiniana* inoculation.

#### Expression of chitinase and glucanase genes

Chitinase (*Chi1*) and glucanase (*Glu1*) genes were up-regulated in response to various foliar treatments 48 h after inoculation, in both pot and field experiments (Fig 7). The combined formulation of JAF2+MFF2 induced the greatest up-regulation of *Chi1* and *Glu1* by 44 and 7-fold, respectively, in the pot experiment and, 39 and 6-fold, respectively in field experiment as compared to the inoculated control. All other botanical-chemical formulations increased in the expression of *Chi1* (9 to 20-fold in the pot and 7 to 18-fold in field experiment) and *Glu1* (2 to 6-fold in the pot and 1 to 4-fold in field experiment) when compared to the untreated inoculated control. Overall, the increase in the level of expression of the selected genes was higher in the pot experiment than in the field.

#### Expression of genes for pathogenesis-related proteins (*PR-*4 and *PR-5*)

All the treatments up-regulated the expression of *PR-4* and *PR-5* genes, although the level of this varied significantly (Fig 8). The combined application of JAF2+MFF2, induced expression of *PR-4* and *PR-5* 48 h after pathogen inoculation by 24 and 25-fold in the pot and 23-fold in the field experiment, respectively, as compared to the inoculated control. All formulations increased the level of expression of *PR-4* (3 to 16-fold in both the pot and field) and *PR-5* (2 to 21-fold in both the pot and field experiments), as compared to the inoculated control.

### Effect of botanical-chemical formulations on wheat yield in field experiments

In the field experiments e mean data of two years (Table 6) gave an increase in no. of grains/spike and yield of uninoculated wheat plants with JAF1 (30 and 13%, respectively) followed by THF1, CAF1 and foliar application of SAF1 (19, 29 and 21%, respectively, increase in no. of grains and 8, 11 and 9%, respectively, increase in yield) as compared to the uninoculated (uninfected) control. However, the same treatments resulted in an increase in 100 grains weight of wheat ranging from 4–12% than the uninoculated control plants.

**Table 6:** Effect of botanical formulations on the yield of wheat inoculated with *B*. *sorokiniana*.

Treatments	No. of grains/spike			100 grains weight			Yield (kg/ha)		
	2010–11	2011–12	Mean	2010–11	2011–12	Mean	2010–11	2011–12	Mean
Control	54.3^i^	57.3^h^	55.8^i^	6.51^def^	6.76^cde^	6.63^defg^	4198^gh^	4302^g^	4250^i^
JAF1	72.0^b^	73.3^b^	72.6^b^	7.16^a^	7.77^a^	7.46^a^	4672^ab^	4777^ab^	4724^b^
THF1	65.3^cde^	67.7^bcde^	66.5^de^	6.77^cd^	7.04^bc^	6.90^cde^	4547^bcd^	4659^bcd^	4603^d^
CAF1	71.3^bc^	72.3^bc^	71.8^b^	7.09^ab^	7.67^a^	7.38^ab^	4671^ab^	4778^ab^	4724^b^
SAF1	66.0^bcd^	68.7^bcde^	67.3^cd^	6.83^bc^	7.07^bc^	6.95^cd^	4603^abc^	4708^abc^	4655^c^
BS	43.7^j^	47.0^i^	45.3^j^	3.19^j^	3.49^i^	3.34^j^	3505^i^	3621^h^	3563^l^
MFF1+BS	63.3^defg^	64.3^defg^	63.8^fg^	6.47^ef^	6.75^cde^	6.61^defg^	3962^gh^	4071^g^	4016^k^
JAF1+BS	62.7^defg^	63.7^efg^	63.2^g^	6.42^f^	6.68^def^	6.55^efgh^	4219^g^	4325^g^	4272^i^
THF1+BS	56.0^hi^	57.3^h^	56.6^i^	5.78^i^	5.97^h^	5.87^i^	4093^h^	4203^g^	4148^j^
CAF1+BS	58.0^ghi^	59.7^gh^	58.8^h^	6.02^hi^	6.39^fg^	6.20^hi^	4217^gh^	4327^g^	4272^i^
SAF1+BS	60.3^efgh^	61.7^fgh^	61.0^h^	6.06^h^	6.28^gh^	6.17^hi^	4285^efg^	4361^efg^	4323^g^
JAF2+MFF2+BS	84.3^a^	86.7^a^	85.5^a^	6.91^abc^	7.14^b^	7.02^bc^	4739^a^	4844^a^	4791^a^
THF2+MFF2+BS	67.7^bcd^	70.0^bcd^	68.8^c^	6.10^gh^	6.45^efg^	6.27^gh^	4401^def^	4508^def^	4454^f^
CAF2+ MFF2+BS	68.0^bcd^	70.7^bcd^	69.3^bc^	6.72^cde^	7.14^b^	6.93^cde^	4434^cde^	4540^cde^	4487^e^
SAF2+MFF2+BS	59.3^g^	61.7^fg^	60.5^ij^	6.46^ef^	7.02^bcd^	6.74^cdef^	4214^gh^	4325^g^	4269^i^
JAF2+SAF2+BS	64.0^def^	66.3^cdef^	65.1^efg^	6.53^def^	6.69^def^	6.61^defg^	4278^efg^	4384^eg^	4331^g^
THF2+SAF2+BS	64.3^def^	67.3^bcdef^	65.8^def^	6.49^ef^	6.74^cde^	6.61^defg^	4240^fgh^	4352^fg^	4296^h^
CAF2+SAF2+BS	62.7^defg^	65.0^cdefg^	63.8^fg^	6.37^fg^	6.61^efg^	6.49^fgh^	4199^gh^	4306^g^	4252^i^
LSD values	5.55	5.78	2.17	0.28	0.34	0.39	174.12	181.59	23.54

Data expressed as ± standard deviation of mean (SD) of three replicates.

Mean values with different letters are significantly different from each other according to the least significant difference (*LSD*) at P< 0.05.

See Table 1 for abbreviations.

Inoculation of wheat plants with *B*. *sorokiniana* exhibited a significant decrease in no. of grains/spike, 100-grains weight and yield of wheat (19, 50 and 16%, respectively) as compared to the uninoculated control at P<0.05. The fungicide MFF1 application significantly increased the no. of grains/spike, 100-grain weight and grain yield by 41, 98 and 12%, respectively, as compared with the infected control. JAF1, THF1, CAF1 and SAF1 increased the no. of grains/spike ranging from 25–39%, grain weight ranging from 76–101% and grain yield by 16, 20 and 21%, respectively, as compared to the infected control. The maximum increase (89, 110 and 34%) in no. of grains/spike, grains weight and grain yield was exhibited by the combined application of JAF2+MFF2.

## Discussion

In this study, we examined the potential of *Jacaranda mimosifolia*, *Thevetia peruviana*, and *Calotropis procera* leaf extracts as foliar application to protect wheat against *B*. *sorokiniana*. Foliar application with these extracts was not only found to be an effective way of controlling leaf blotch disease but also reduced the required dose of synthetic fungicide.

Firstly, we observed that the selected plant extracts effectively inhibited the growth of *B*. *sorokiniana in vitro* using terbinafine as standard fungicide positive-control (Table 4). Our colleague Hossain et al [38], had demonstrated the *in vitro* inhibitory effects of neem leaf extracts and garlic clove against *B*. *sorokiniana*. *Vernonia amygdalina* crude extracts exhibited antifungal potential and its compounds like tannins, saponins and glycosides likely to be responsible for the fungitoxicity [39]. In addition, *Thymus vulgaris* and *Zingiber officinale* extracts are also reported to inhibit the mycelial growth of phytopathogenic fungi [40,41].

Then we observed that foliar application of selected plant extracts particularly *J*. *mimosifolia* augmented the effectiveness of mefenoxam, as the JAF2+MFF2 gave significantly higher levels of inhibition of leaf spot blotch than either the JAF1 or MFF1. The principal inhibitory effects were observed on leaf blight in wheat infected with *B*. *sorokiniana* in the pot as well as in the field. The combined application of JAF2+MFF2 was found to be much more effective in inoculated wheat plants rather than other treatments in both pot and field experiments. Foliar application of JAF1, JAF2+MFF2 and JAF2+SAF2 were most effective to inhibit the spot blotch infection by 77–86% followed by the combined treatment THF2+MFF2 in both the pot and field experiments. The JAF1 was 2% more effective in reducing disease in pot experiment than in field experiment where other environmental factors might be one of the reasons to modify the effects of biofungicide in addition to the plethora of microbes in the rhizosphere. However, combined applications of JAF2+MFF2 and JAF2+SAF2 exhibited the same magnitude of diseases reduction in both pot and field experiment. Plant extract further augmented the efficiency of fungicide and SA with similar magnitude, as the mixed formulation of JAF2+MFF2 and JAF2+SAF2 showed statistically significant 10–20% greater inhibition against spot blotch disease than the plant extract and chemical fungicide applied alone. This potential inhibitory effect of the plant extracts as a potent biofungicide was in agreement with the similar findings of our colleagues [42], who reported the inhibitory effects of *Z*. *jujuba* and *I*. *carnea* leaf extracts against sheath blight disease of rice. The maximum reduction in mycelial growth by plant extract might be due to the antifungal compounds present in the plant extract. Foliar application of plant extracts has also been reported to control the early blight infection of tomato plants [43].

To expand our knowledge of the wheat- *B*. *sorokiniana* interaction in response to different foliar treatments with *J*. *mimosifolia*, *T*. *peruviana*, *C*. *procera* extracts, total soluble protein, phenolic content, defense-related enzymes and PR proteins were analyzed. Different treatments with selected plant extracts applied alone, as well as in combination with fungicide and SA, significantly increased the total chlorophyll, leaf soluble protein, phenolic content and activities of defense-related enzymes as compared with the inoculated control, and chemical fungicide treatment in the pot experiment (Figs 2, 3, 4, 5 & 6). In the field experiment, despite environmental effects, all the treatments gave an increase in photosynthetic pigments, protein, phenolic content, defense-related enzyme activities and grain yield in response to *B*. *sorokiniana* infection (Figs 2, 3, 4, 5 & 6; Table 5). These findings are in accordance with those of Kamalakannan, et al. [44], who reported the efficacy of foliar application of leaf extract of *Prosopis juliflora* to reduce the blast infection in field experiments besides increasing the crop yield. In both pot and field experiments, the combination that gave the highest significant increase in photosynthetic pigments, total protein, phenolic content and defense enzymes was JAF2+MFF2 followed by JAF2+SAF2. The mentioned half-strength treatment provided defense in wheat plants against *B*. *sorokiniana* by inducing the defense related enzymes and up-regulating the expression of pathogenesis-related proteins, which may play an important role in strengthening the cell walls of the host plant to resist the *B*. *sorokiniana* infection. These findings reveal the host-pathogen interactions underlying defense/resistance in response to *J*. *mimosifolia* applications. Exogenous foliar application of selected plant extracts induced resistance in the host plant via induction of higher levels of host defense enzymes. This might be due to the presence of active compounds in the extracts which induced systemic resistance in the host plants, resulting in reduction of disease development [45]. Our colleagues [46], reported the increased activities of peroxidase, polyphenol oxidase and catalase enzymes in powdery mildew infected leaves, and induced systemic resistance in the host plant which support the findings of current study. Induced resistance in plants may increases the ability of susceptible plants to withstand pathogens in a non-genetic way [47]. It is reported in the previous studies that the induced resistance prior to challenge infection elevates the level of some defense compounds and sensitizes the plants to rapidly produce some compounds after infection and thereby, provide protection against the disease [48]. The onset of induced systemic resistance in plants correlates with the increased activity of defense related enzymes and PR proteins such as polyphenol oxidase, chitinases, β-1,3-glucanases, PAL and peroxidases; consequently, PR proteins are generally used as ISR markers [49, 50].

## Conclusion

In conclusion, this study has demonstrated that the foliar application of *J*. *mimosifolia* leaf extract effectively controlled leaf blight disease under *in vivo* conditions. The combined half-strength application of *J*. *mimosifolia* leaf extracts with fungicide (JAF2+MFF2) lowered the dose of chemical fungicide (mefenoxam) required while inducing resistance in wheat to subsequent inoculation with *B*. *sorokiniana*, apparently through the accumulation and up-regulation of defense-related enzymes and PR-proteins. The cost of fungicide applied to control leaf blight disease is $11/ha (calculation based on the field experiment conducted here), while JAF2+MFF2 replaces 50% of the cost of the fungicide. As *J*. *mimosifolia* is a naturally growing tree and its leaf extract preparation may cost only approximately $1–2.3/ha. Hence, the combined application of *J*. *mimosifolia* leaf extracts with available synthetic fungicide is highly promising. We believe that such systemic fungicides could be sold commercially in pre-pack mixtures with contact fungicides. Moreover, the application of such botanical-chemical formulations not only indirectly reduced the side effects of chemical fungicides but also decreased their residual effects on the human health and environment.

## Supporting information

S1 Text Table A. Gene-specific primers used for RT-qPCR analysis. (DOCX)

S1 Dataset Pot & field experiment data. (XLSX)
