# Supplementary material for: Botanical-chemical formulations enhanced yield and protection against Bipolaris sorokiniana in wheat by inducing the expression of pathogenesis-related proteins
Source: PLoS One. 2018 Apr 30;13(4):e0196194. doi: 10.1371/journal.pone.0196194 (PMC5927443; doi:10.1371/journal.pone.0196194)
Supplement: S1 Text — (DOCX) [file pone.0196194.s001.docx]

| **Gene** | **Forward primer (5'-3')** | **Reverse primer (5'-3')** | **Amplicon size (bp)** | **Tm (◦C)** | **Gene Description** | **Reference** |
| --- | --- | --- | --- | --- | --- | --- |
| Chi1 | gggctactgcttcaaggaaga | acactaggtctgggttgctca | 146 | 60;59 | class 1 basic chitinase | Z.-X. Lu., et al 2006 |
| Glu1 | acgtacattttcgccatgttc | cacagacagacgagtggttga | 154 | 60;59 | acidic β 1-3 glucanase | Z.-X. Lu., et al 2006 |
| PR4 | acaccgtcttcaccaagatcgaca | agcatggatcagtctcagtgctca | 190 | 60;59 | chitinase | Qi, P.F. et al., 2012 |
| PR-5 | atcaccaaggattgcctcaa | gtgaaggtgctggtctggtc | 196 | 60;60 | thaumatin-like protein | Roberti, R. et al 2008 |
| Actin | ggaaaagtgcagagagacacg | tacagtgtctggatcggtggt | 150 | 60;60 | Actin (TaBs109G1 clone) | Z.-X. Lu., et al 2006 |

**Table A S1 Table Gene-specific primers used for RT-qPCR analysis**
